# Assessing the genetic diversity of cowpea [*Vigna unguiculata* (L.) Walp.] germplasm collections using phenotypic traits and SNP markers

**DOI:** 10.1186/s12863-020-00914-7

**Published:** 2020-09-18

**Authors:** Nelia Nkhoma, Hussein Shimelis, Mark D. Laing, Admire Shayanowako, Isack Mathew

**Affiliations:** 1 African Centre for Crop Improvement, University of KwaZulu-Natal, P/Bag X01, Scottsville, Pietermaritzburg, 3209 South Africa; 2 Seed Control and Certification Institute, P. O. Box 350199, Chilanga, Lusaka, Zambia

**Keywords:** Cowpea, Genotypic diversity, Phenotypic traits, SNP makers, Population structure, Yield components, Zambia

## Abstract

**Background:**

Productivity of cowpea [*Vigna unguiculata* (L.) *Walp*] in sub-Sahara Africa is curtailed by a lack of farmer-preferred and improved cultivars and modern production technologies. The objectives of the study were to determine the extent of genetic diversity present among a collection of cowpea accessions from Zambia and Malawi using phenotypic traits and single nucleotide polymorphism (SNP) markers and, to select distinct and complementary parental lines for cultivar development. One hundred cowpea genotypes were evaluated for agronomic traits in two selected sites in Zambia, using a 10 × 10 alpha lattice design with two replications. Ninety-four of the test genotypes were profiled with 14,116 SNP markers.

**Results:**

Number of pods plant^− 1^ (NPP), pod length (PDL), and number of seeds pod^− 1^ (NSP), were significantly (*p* < 0.05) affected by genotype × environment interaction effects. Genotypes such as CP411, CP421, CP645, CP732, Chimponongo, and MS1–8–1-4 exhibited higher grain yield of > 1200 kg/ha with excellent performance in yield components such as NSP, PDL, HSW and GYD. Grain yield had significant (*p* < 0.05) associations with NPP (*r* = 0.50), NSP (*r* = 0.46) and PDL (*r* = 0.42) useful for simultaneous selection for yield improvement in cowpea. The SNP markers revealed gene diversity and polymorphic information content of 0.22 and 0.17, respectively, showing that the tested cowpea accessions were genetically diverse. Test genotypes were classified into four genetic groups irrespective of source of collection allowing selection and subsequent crosses to develop breeding populations for cultivar development.

**Conclusions:**

Genotypes Bubebe, CP411, CP421, CP645, Chimponogo and MS1–8–1-4 were identified to be the most genetically divergent and high yielding making them ideal parental lines for breeding. This study provided a baseline information and identified promising cowpea genetic resources for effective breeding and systematic conservation.

## Background

Cowpea [*Vigna unguiculata* (L.) Walp., 2n = 2x = 22] is a relatively low cost source of plant-derived protein, amino acids and essential nutrients globally. It is the main food staple supporting millions of people in sub-Sahara Africa (SSA) [1, 2]. The grain protein content of cowpea is about 250 mg/g [3], which is comparable to that of soybeans [2]. In addition, cowpea grain contains essential nutrients such as iron (53.2 mg/kg), zinc (38.1 mg/kg), calcium (826 mg/kg) and magnesium (1915 mg/kg) [3]. Young and succulent leaves and pods of cowpea are used as cooked vegetable, while the grains are ground and processed into powder for making thick porridge, gravy or sometimes consumed as a boiled delicacy [4].

Cowpea is a key companion crop in mixed cropping systems useful to supressing weed infestation, enhancing soil fertility and reducing water evaporation [5]. Cowpea forms symbiosis with the root nodule bacterium, *Rhizobium*, and fixes 70 to 350 kg/ha of atmospheric nitrogen and some 40 to 80 kg of this is deposited into soils as a natural source of mineral nitrogen contributing to soil health [5]. Cowpea thrives under low soil fertility and dry-land growing conditions making it one of the most resilient legume crops suitable for the low input and water-limited production systems in SSA.

Global production of cowpea is estimated to be 6.5 million tons per annum on 14.5 million hectares of land [6]. The leading world producers of cowpea are Nigeria and Niger with five and three million hectares of production areas, respectively [7]. Cowpea is widely cultivated by small-scale farmers in southern African countries such as in Zambia, Zimbabwe, Malawi, Namibia, Mozambique and Botswana [8, 9]. The mean grain yields of cowpea in SSA is between 100 to 599 kg/ha which is far less than the potential yield of the crop reaching up to 3 t/ha elsewhere [8, 10]. The yield gap is attributable to a lack of improved and high yielding cultivars, poor agronomic practices and an array of abiotic and biotic production constraints. Therefore, there is need to develop best performing, locally adapted and farmer-preferred cowpea varieties for sustainable production in the region.

The southern African countries including Namibia, Botswana, Zambia, Zimbabwe, Malawi, Mozambique and South Africa are believed to be the centres of diversity of cowpea where primitive and wild relatives are found [11]. Diverse cowpea germplasm collections are conserved in the Southern African Development Community (SADC) gene bank in Lusaka/Zambia. The country serves as Plant Genetic Resources Centre coordinating the works of some 16 National Plant Genetic Resources Centres (NPGRCs) in southern Africa [12]. Farmers in southern Africa widely grow unimproved landraces due to a lack of improved and locally adapted farmer-preferred cultivars. Landraces exhibit low yield potential, heterogeneous in flowering and maturity, poor processing quality, and low palatability and digestibility [13]. Low palatability and digestibility are adaptive traits against field and storage pests, traits resulted from repeated cycles of natural and artificial selection. The low palatability and digestibility of landraces reduce their utility for human consumption due to prolonged cooking time and reduced bioavailability of essential nutrients. Therefore, the cowpea genetic resources found in the region can be explored as a novel source of genetic variation for breeding programs.

A well-characterised crop genetic resource is a precondition for effective breeding and genetic conservation. Genetic diversity is assessed using phenotypic traits and molecular markers. Phenotypic characterisation in the target production environment enables identification and quantification of genetic variation for key qualitative and quantitative traits for ideotype breeding. Knowledge of phenotypic variation and traits relationship assist crop breeders to develop the most adaptive and productive cultivars [14]. The genetic diversity of cowpea for phenotypic traits is assessed using standard descriptors developed by the International Board for Plant Genetic Resource [15]. Key phenotypic traits include days to flowering, time to maturity, growth habit, flower colour, number of pod plant^− 1^, pod length, number of seeds pod^− 1^, seed colour, seed size, hundred seed weight and grain yield [15].

Various DNA markers such as the restriction fragment length polymorphism (RFLP), amplified fragment length polymorphism (AFLP), simple sequence repeat (SSR), random amplified polymorphic DNA (RAPD) and single nucleotide polymorphisms (SNP) have been used in cowpea genetic diversity analysis [16–18]. SNPs are markers of choice in genetic diversity analysis because they are widely distributed throughout the genome and their detection is amenable to automation [19]. In addition, SNP markers are increasingly time and cost efficient to genotype large populations with a relatively higher throughput [20]. SNP markers were applied in genetic diversity analysis of cowpea [16].

Cowpea is one of food security crops in Zambia widely cultivated in the eastern, southern and western regions. Hitherto, only seven cowpea varieties were released in the country that are relatively poor performers (< 700 kg/ha) and largely succumbed to emerging pests and diseases. The genetic diversity present among the germplasm collections conserved in the gene bank and landraces cultivated by smallholder farmers in Zambia can be explored for cowpea breeding and new cultivar deployment. Therefore, the objectives of the present study were to determine the extent of genetic diversity present among a collection of cowpea accessions from Zambia and Malawi using phenotypic traits and SNP markers, and to select distinct and complementary parental lines for cultivar development in Zambia.

## Results

### Analysis of variance based quantitative phenotypic traits across locations

The combined analysis of variance revealed that the genotype × site interaction effects were significant (*p* < 0.05) for PDL, NPP and NSP (Table 1). DTF, DTM, PDL and NPP varied significantly (*p* < 0.05) between the two sites. The genotypes had varied flowering and maturity date as revealed by the significant (*p* < 0.05) genotypic effect. Similarly, there was significant (*p* < 0.05) genotype difference for PDL, NSP, HSW and GYD.

**Table 1:** Mean squares and significant tests among 100 cowpea germplasm collections evaluated based on eight quantitative agronomic traits in two locations in Zambia

Source of variation	DF	DTF	DTM	PDL	NPP	NSP	SDS	HSW	GYD
Location (L)	1	702.20*	13,806.30***	542.61***	2550.25***	29.7	0.01	6.30	107,770.00
Rep(R)	2	8.30	1731.08***	56.94**	20.91	1.72	2.40	0.67	388,357.00
Block (B)	18	196.90	167.30	8.84	61.44	14.77*	5.09*	13.25*	1,070,734.00***
Genotype (G)	99	242.50*	154.70*	14.02*	64.44	11.93*	2.10	10.26*	532,280.00***
Genotype × location	99	161.00	124.30	15.13*	71.32*	11.24*	1.06	8.15	233,499.00
Residual	180	148.00	107.20	10.66	50.41	8.66	1.72	7.07	207,464.00
Total	399	177.60	168.40	14.08	65.69	10.41	1.80	8.38	334,119.00

Note: *, **,*** = Significance at 10, 5 and 1%, respectively; *ns* not significant, *DF* degrees of freedom, *DTF* days to flowering, *DTM* days to maturity, *PDL* pod length (cm), *NPP* number of pods per plant, *NSP* number of seeds per pod, *SDS* seed size, *HSW* hundred seed weight (g) and *GYD* grain yield in kg per hectare

### Mean performance of cowpea genotypes

The mean days to flowering of the test genotypes was 41 days. DTF varied from 22 days (for the genotype BB10–4–2-5) to 59 days (Kapita black) (Table 2). The mean DTM of test genotypes was 74 days. Genotype ZM2960 was relatively early maturing with 60 days to maturity. Other early maturing genotypes included BB10–4–2-5 (62 days), Lutechipata and ZM6680 (63 days). The number of pods per plant varied from 13 to 33. Genotypes MS1–8–1-4, CP411, BBXSC103 and Kapita black had the highest NPP (> 30 pods plant^− 1^). Pod length varied amongst genotypes. The longest pod were recorded for BBXSC13 and MS1–8–1-4 with a mean of 21 cm. The genotypes that recorded higher number of seeds per pod were Bubebe, CP421 and CP 3422 with 18.50, 18.25 and 18.25 seeds per pod, respectively.

**Table 2:** Mean values for grain yield and yield components of 100 cowpea genotypes showing the top 10 and bottom 5 ranked genotypes based on grain yield (kg/ha) when assessed in two locations in Zambia

Genotype	DTF	DTM	PDL	NPP	NSP	SDS	HSW	GYD
Top 10 genotypes								
CP411	34.50	73.75	20.15	32.25	16.50	3.00	13.48	2197.70
Chimponongo	51.50	79.50	19.52	26.75	16.75	5.00	20.95	2093.20
CP645	51.00	73.25	20.68	28.00	17.25	4.50	13.48	1899.30
MS1–8–1-4	39.75	68.50	21.20	33.25	15.50	5.00	15.03	1779.80
CP732	34.75	81.00	17.02	22.25	16.00	4.50	15.50	1672.40
BB14–16–2-2	36.75	74.00	19.70	25.25	15.50	3.00	11.25	1501.90
ZM3003	39.00	74.50	16.68	18.50	13.50	6.00	14.08	1454.10
CP421	44.75	72.75	19.90	24.00	18.25	3.00	16.23	1328.20
CP2	39.50	75.00	17.53	26.75	16.25	4.50	11.75	1252.70
CP601	40.00	73.50	17.85	22.25	16.75	5.50	13.83	1237.80
Bottom 5 genotypes								
ZM2966	38.50	74.50	17.25	18.75	13.75	4.00	13.43	227.10
CP2231	45.75	77.00	15.41	16.75	12.75	6.00	15.13	225.40
ZM2954	47.00	73.75	15.90	19.25	13.50	5.50	14.68	188.20
CP1769	35.25	73.00	18.63	21.25	17.25	5.00	13.55	126.00
ZM6680	29.25	62.75	12.30	16.50	11.25	5.00	15.55	87.00
Mean	41.10	73.86	17.98	21.40	15.60	4.20	12.93	748.56
Minimum	14.00	31.00	6.00	3.00	0.00	3.00	9.10	32.55
Maximum	80.00	96.00	27.20	56.00	24.00	7.00	28.50	5471.24
SE	8.60	7.32	2.31	5.02	2.08	0.92	1.88	322.10
LSD (5%)	16.97	14.50	4.56	9.90	4.11	1.83	3.71	635.50
CV (%)	29.60	14.02	18.16	33.18	18.86	31.09	20.55	60.85

Note: *CV* coefficient of variation, *LSD* least significant difference, *SE* standard error, *DTF* days to flowering, *DTM* days to maturity, *PDL* pod length (UNIT?), *NPP* number of pods per plant, *NSP* number of seeds per pod, *SDS* seed size (mm), *HSW* hundred seed weight (g/100 seed), *GYD* grain yield in kg ha^− 1^

Heavier hundred seed weight was recorded for the genotypes Kapita (15.95 g/100 seed), CP2980 and ZM6680 (15.55). There existed significant genotype difference for GYD ranging from 87 kg ha^− 1^ (for genotype ZM 6680) to 2197.7 kg ha^− 1^ (CP411). The overall mean GYD of test genotypes was 748.56 kg ha^− 1^. Genotypes Chimponongo (with mean GYD of 2093.2 kg ha^− 1^), CP645 (1899 kg ha^− 1^) and MS1–8–1-4 (1779.80 kg ha^− 1^) were among the top yielding selections. Overall, the following test genotypes were selected: Bubebe, BBXSC13, Chimponongo, CP411, CP645 and MS1–8–1-4 based on suitable and complementary quantitative agronomic traits. These genotypes are recommended as breeding parents to develop cowpea-breeding populations.

### Variation based on qualitative phenotypic traits

There were significant differences (*P* < 0.00) among test genotypes for key qualitative traits (Additional file 1). For growth habit, 43 of the accessions were indeterminate, 39 determinate and 18 creeping types. Genotypes with predominantly upright growth type and short plant height were Bubebe, Namuseba, Msandile and MS1–8–1-4. Chimponongo and BBXSC13 had creeping growth type. Forty-nine accessions had brown and 21 black seed coat colour, while the rest of the genotypes had 12 purple- brown, 10 white and 8 red- brown. Based on leaf colour genotypes were assorted into light green (26 genotypes), light green (35) and dark green (39). Pod colour was variable varying from deep green (52 genotypes), light green (30) and purple (18). There were three classes of genotypes based on flower colour: 95 genotypes displayed violet flower, while four had yellow and one had white. Therefore, a combination of the assessed qualitative traits are useful markers for genotype selection in cowpea improvement programs.

### Variance components and heritability of quantitative agronomic traits

Phenotypic coefficient of variation (PCV) values were higher than genotypic coefficient of variation (GCV) for all the traits (Table 3). The GCV values ranged from 0 to 14.6%, while the PCV ranged from 0 to 21.56%. Larger discrepancies between GCV and PCV estimates were observed for all assessed traits. The genotypic variance accounted for ≥50% of the total variation for grain yield. Low heritability (≤ 30) estimates were recorded for days to maturity, hundred seed weight, number of seed pod^− 1^ and pod length and number of pod plant^− 1^. The heritability estimates for days to flowering and seed size were moderate (30–60%), while grain yield recorded heritability estimates above 60% that will enhance the response to selection and breeding gains. Genetic advance ranged from 0 to 20.58%. Seed size and days to flowering had moderate GA% (10–20%).

**Table 3:** Estimates of variance components and genetic parameters for yield and yield components among 100 cowpea genotypes evaluated in two locations in Zambia

Component	DTF	DTM	PDL	NPP	NSP	SDS	HSW	GYD
Genotype (G)	21.75	9.27	0.00	0.00	0.42	0.38	0.57	0.10
Location (L)	148.31	125.56	11.10	50.46	8.65	1.78	7.48	0.23
G x L	6.32	0.00	2.01	10.43	1.30	0.00	0.34	0.00
Total (G + L + G x L)	176.38	134.83	13.12	60.89	10.37	2.17	8.39	0.34
Phenotypic variance	61.99	40.66	3.78	17.83	3.23	0.83	2.61	0.16
Heritability (%)	35.00	23.00	0.00	0.00	13.00	46.00	22.00	64.00
GCV (%)	11.35	4.12	0.00	0.00	4.14	14.68	5.82	0.04
PCV (%)	19.16	8.63	10.82	19.73	11.52	21.58	12.48	0.05
GA	5.69	2.99	0.00	0.00	0.48	0.87	0.72	0.53
GA (%)	13.85	4.05	0.00	0.00	3.07	20.58	5.60	0.07

*GCV* genotypic coefficient of variation, *PCV* phenotypic coefficient of variation, *GA* genetic advance, *GA (%)* genetic advance as a percentage of the mean, *DTF* days to flowering, *DTM* days to maturity, *PDL* pod length, *NPP* number of pods per plant, *NSP* number of seeds per pod, *SDS* Seed Size, *HSW* hundred seed weight (g), *GYD* grain yield in kg per hectare.

### Correlations among quantitative traits

Phenotypic correlation coefficients among assessed quantitative traits is summarised in Table 4. Grain yield showed significant (*P* ≤ 0.05) correlations with PDL (*r* = 0.42), NPP (r = 0.50) and NSP (r = 0.46). The following traits exhibited significant (*P* ≤ 0.05) correlations: DTF and DTM (r = 0.66), PDL with NPP (r = 0.44) and NSP (r = 0.64). NPP and NSP were significantly correlated (r = 0.38), while HSW and SDS exhibited a relatively stronger association (r = 0.51).

**Table 4:** Correlation coefficients of grain yield and yield components among 100 cowpea genotypes evaluated at two locations in Zambia

Traits	DTF	DTM	PDL	NPP	NSP	SDS	HSWT	GYD
**DTF**	1							
**DTM**	0.66**	1						
**PDL**	− 0.05	0.01	1					
**NPP**	−0.05	− 0.05	0.43**	1				
**NSP**	−0.05	0.03	0.64**	0.38**	1			
**SDS**	−0.01	0.00	−0.04	− 0.09	−0.30**	1		
**HSW**	0.01	−0.06	0.07	−0.09	−0.12	0.51***	1	
**GYD**	−0.05	−0.07	0.42**	0.50**	0.46**	−0.12	0.04	1

Note: **.*** = Significant at 5 and 1% respectively; *DTF* days to flowering, *DTM* days to maturity, *PDL* pod length (cm), *NPP* number of pods per plant, *NSP* number of seeds per pod, *HSW* hundred seed weight (g), *GYD* grain yield in kg per hectare

### Principal component (PC) and bi-plot analyses

The first three PCs with Eigen-values greater than 1 accounted for 71.25% of the total variation exhibited by the assessed quantitative traits (Table 5). The first principal component (PC1) accounted for 31.5%, while PC2 and PC3 contributed to 20.97 and 18.78%, respectively, of the total variation. The highest contributing traits correlated with PC1 were PDL (0.84), NSP (0.82), GYD (0.75), and NPP (0.72). The loadings on PC2 were mostly contributed by DTF (0.84) and DTM (0.87), while HSW (0.80) and SDS (0.78) had the largest contributions to the variation correlated with PC3.

**Table 5:** Eigen values, variances and loading scores of eight quantitative traits among 100 cowpea genotypes assessed in two locations in Zambia

Traits	PC1	PC2	PC3
Eigen-values	2.52	1.68	1.50
Proportion variance (%)	31.49	20.97	18.78
Cumulative variance (%)	31.49	52.46	71.25
DTF	−0.09	0.88	−0.22
DTM	−0.03	0.87	−0.27
PDL	0.84	0.06	0.04
NPP	0.72	0.02	0.13
NSP	0.82	−0.01	−0.21
SDS	−0.25	0.18	0.78
HSW	0.02	0.31	0.80
GYD	0.75	0.11	0.27

*DTF* days to flowering, *DTM* days to maturity, *PDL* pod length (cm), *NPP* number of pods per plant, *NSP* number of seeds per pod, *HSW* hundred seed weight (g), *GYD* grain yield in kg per hectare, *PC* principal component.

**Fig. 1:**
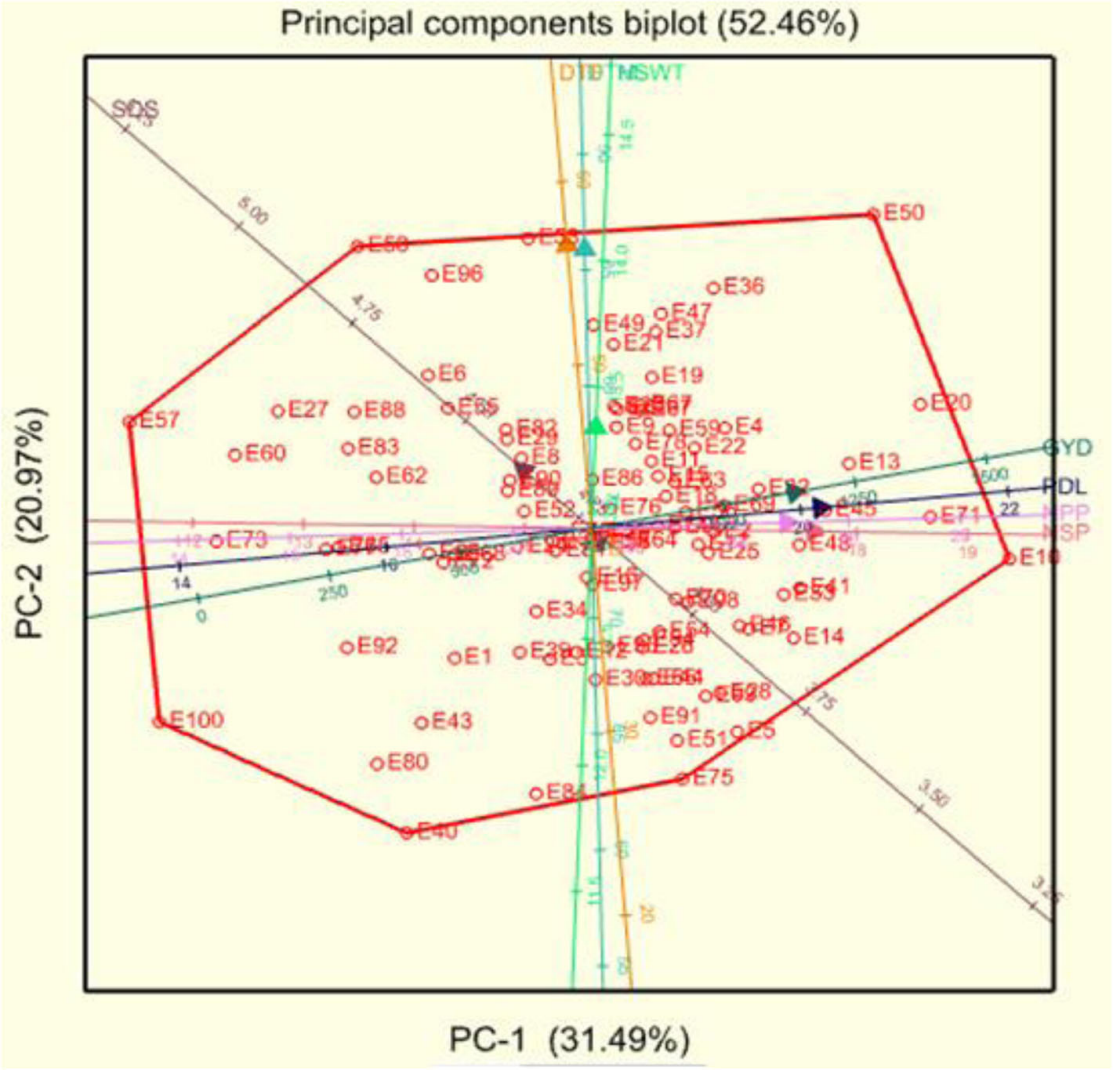
Genotype-trait biplot showing association of eight quntitative traits in 100 genotypes of cowpea assessed in two loactions. Note: DTF: days to flowering; DTM: days to maturity; PDL: pod length (cm), NPP: number of pods per plant; NSP: number of seeds per pod, HSW: hundred seed weight (g), GYD: grain yield in kg per hectare PC-1 and PC-2: principal component 1 and principal components 2, respectively

The relationships among the different traits and genotypes and their association with the respective principal components are further illustrated by the principal component biplot presented in Fig. 1. The biplot dimension vectors showed a high positive correlation among traits GYD, NPP, NSP and PDL, as well as among DTF, DTM, HSW, and SDS. Most of the tested accessions were scattered in the positive side of the first principal component, with genotypes E10 (CP411), E71 (LT16–7–2-5), E13 (CP421) and E20 (CP645) excelling in grain yield and yield components.

### Genetic diversity and population structure

The tested SNP markers were moderately polymorphic with a mean PIC value of 0.17 for the whole population and 0.21 for the mutant lines (Table 6). The mutant lines also exhibited the highest gene diversity (GD) with a mean of 0.26. The highest mean minor allele frequency was 0.22 observed among landraces. The whole population had high heterozygosity (0.30). Among the biological types, mutant lines exhibited the highest heterozygosity estimate with a mean of 0.35. The heterozygosity values fell within a range of 0.25 and 0.36. The elite lines exhibited the highest inbreeding index of − 0.37 while the landraces and mutant lines had indices of − 0.34 and − 0.35, respectively. Overall, the level of inbreeding ranged between − 0.52 and − 0.13.

**Table 6:** Genetic parameters of 90 cowpea germplasm collections assessed based on 14,116 SNP markers

Biological type	GD	PIC	MAF	Ho	F
Whole population	0.22	0.17	0.18	0.30	−0.35
Elite lines	0.21	0.17	0.19	0.30	−0.37
Landraces	0.22	0.18	0.22	0.23	−0.34
Mutant lines	0.26	0.21	0.18	0.35	−0.35
Range	0–0.5	0–0.38	0–0.5	0.25–0.36	−0.52 - -0.13

*GD* genetic diversity, *PIC* polymorphic information content, *MAF* minor allele frequency, *Ho* observed heterozygosity, *F* inbreeding coefficient.

**Fig. 2:**
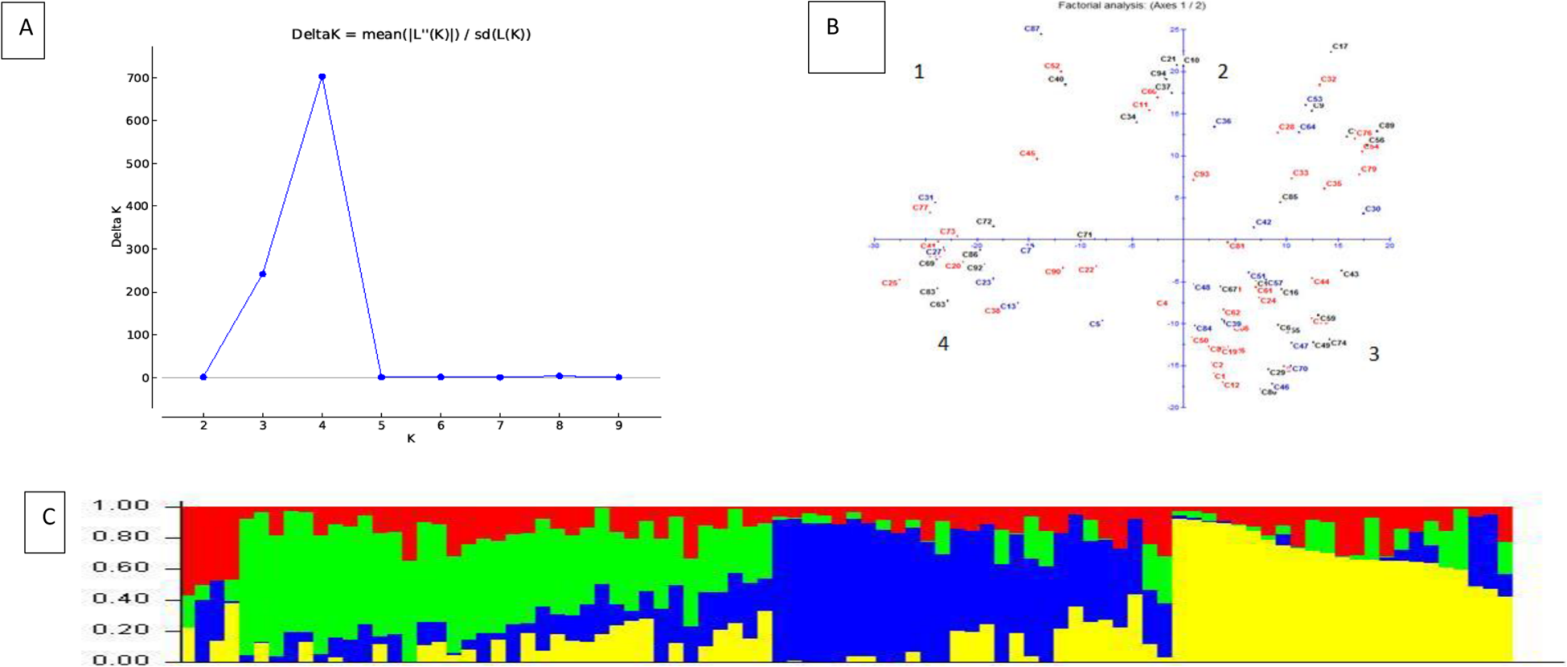
Subpopulation inference among the 90 cowpea accessions based on 14,166 SNPs showing **a** likelihood and delta K values for different number of assumed clusters, **b** principal coordinate analysis clustering of the genotypes and **c** population structure at K = 4

The structure analysis based on the Evanno method allocated the test genotypes into four main clusters with the highest value of ΔK that occurred at K = 4 (Fig. 2 a). Genotypes that scored < 0.80 were considered as pure line populations, while those that were < 0.80 as admixtures (Fig. 2 a). The model-based clustering using the 90 accessions showed the four admixture sub-populations (Fig. 2 c). Sub-population I was composed of 16 accessions (17.7%) that were sourced from Malawi and the University of Zambia. About 22 accessions (24.4%) were allocated in sub-population II and these genotypes were mainly acquired from Malawi, the National Gene Bank of Zambia and the University of Zambia. Sub-population III was the largest group, consisting of 35 accessions (38.9%). Members of this sub-population were landraces and elite lines sourced from the National Gene Bank, and the University of Zambia. Sub-population IV consisted of 17 accessions (18.9%) obtained from the University of Zambia and the National Gene Bank. The sub-population II (University of Zambia) and III (National Gene Bank) were characterized by mean Fst values of 0.57 and 0.69, respectively. Principal coordinate analysis (PCoA) assigned the accessions to four admixture groups. In particular, sub-populations I and II were clustered in PC1, while sub-populations III and IV were dominant in PC2 (Fig. 2b).

The analysis of molecular variance (AMOVA) showed a significant variation within populations. The within population variation accounted for 92% of the total variance (Table 7). The variation detected among the populations was not significant and explained only 8% of the variability in the germplasm. The lack of genetic variation among the populations was confirmed by the low pair-wise genetic differentiation (Fst) values ranging between − 0.004 and 0.012 and inbreeding coefficient (Fis) of − 0.351 to − 0.362 (Table 8).

**Table 7:** Analysis of molecular variance involving 90 cowpea accession based on source of collection

Source	Df	SS	MS	Estimated Variance.	Proportion of variance
Among Populations	2	45.57	22.78	0.54	8%
Within Populations	82	537.96	6.56	6.40	92%
Total	84	583.53		6.95	

*Df* degrees of freedom, *SS* sum of squares, *MS* mean squares.

**Table 8:** Genetic differentiation (Fst) and inbreeding coefficients (Fis) among elite lines, landraces and mutant lines of cowpea evaluated in this study

Populations	Inbreeding coefficient (Fst)		
	G1	G2	G3
G1	–	0.006	0.012
G2	−0.364	–	− 0.004
G3	−0.374	− 0.351	–
Genetic differentiation (Fis)			

*G1* includes all breeding elite lines, *G2* is comprised of landraces collected from farmers in Zambia, *G3* consists of mutant lines.

### Combined analysis of phenotypic and genotypic data

**Fig. 3:**
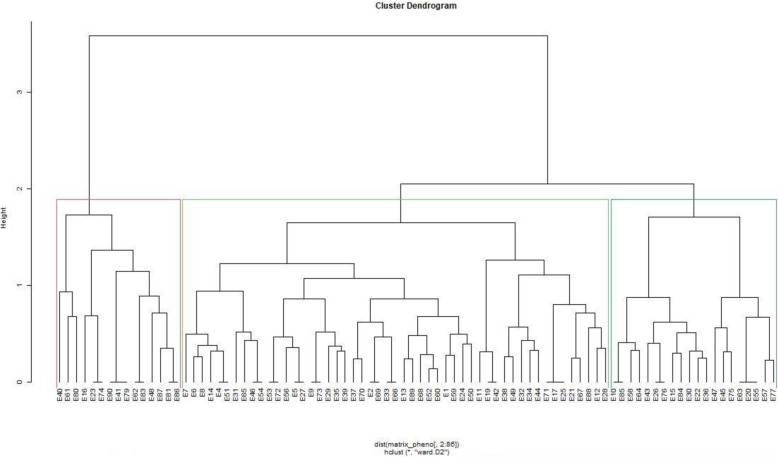
Dendrogram showing relatedness among the 90 cowpea genotypes based on phenotypic matrix

**Fig. 4:**
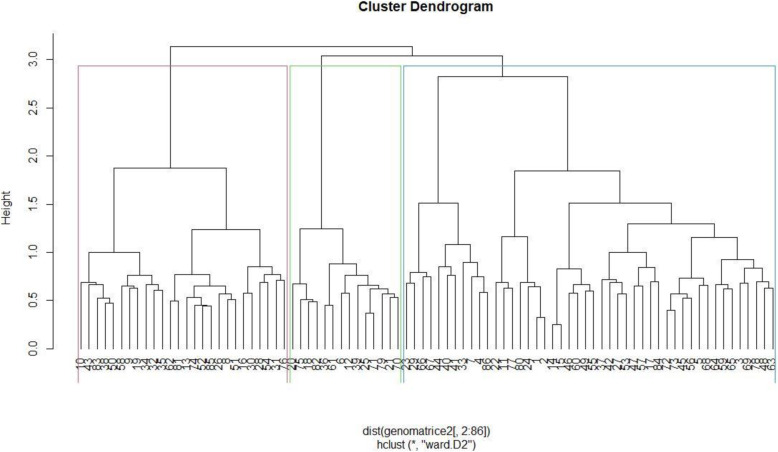
Dendrogram showing relatedness among the 90 cowpea genotypes based on genotypic matrix

**Fig. 5:**
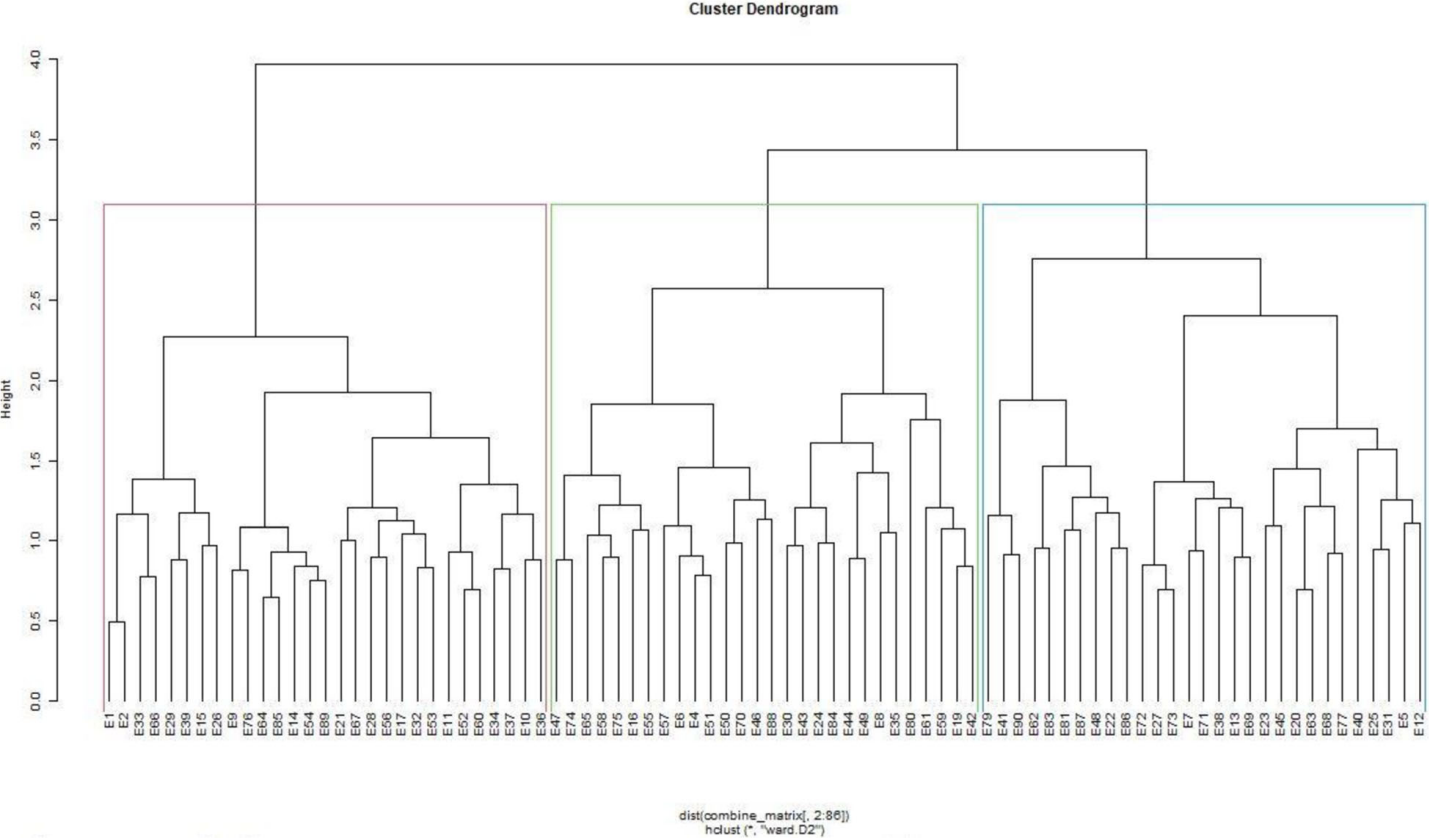
Dendrogram showing relatedness among the 90 cowpea genotypes based on joint matrix

**Fig. 6:**
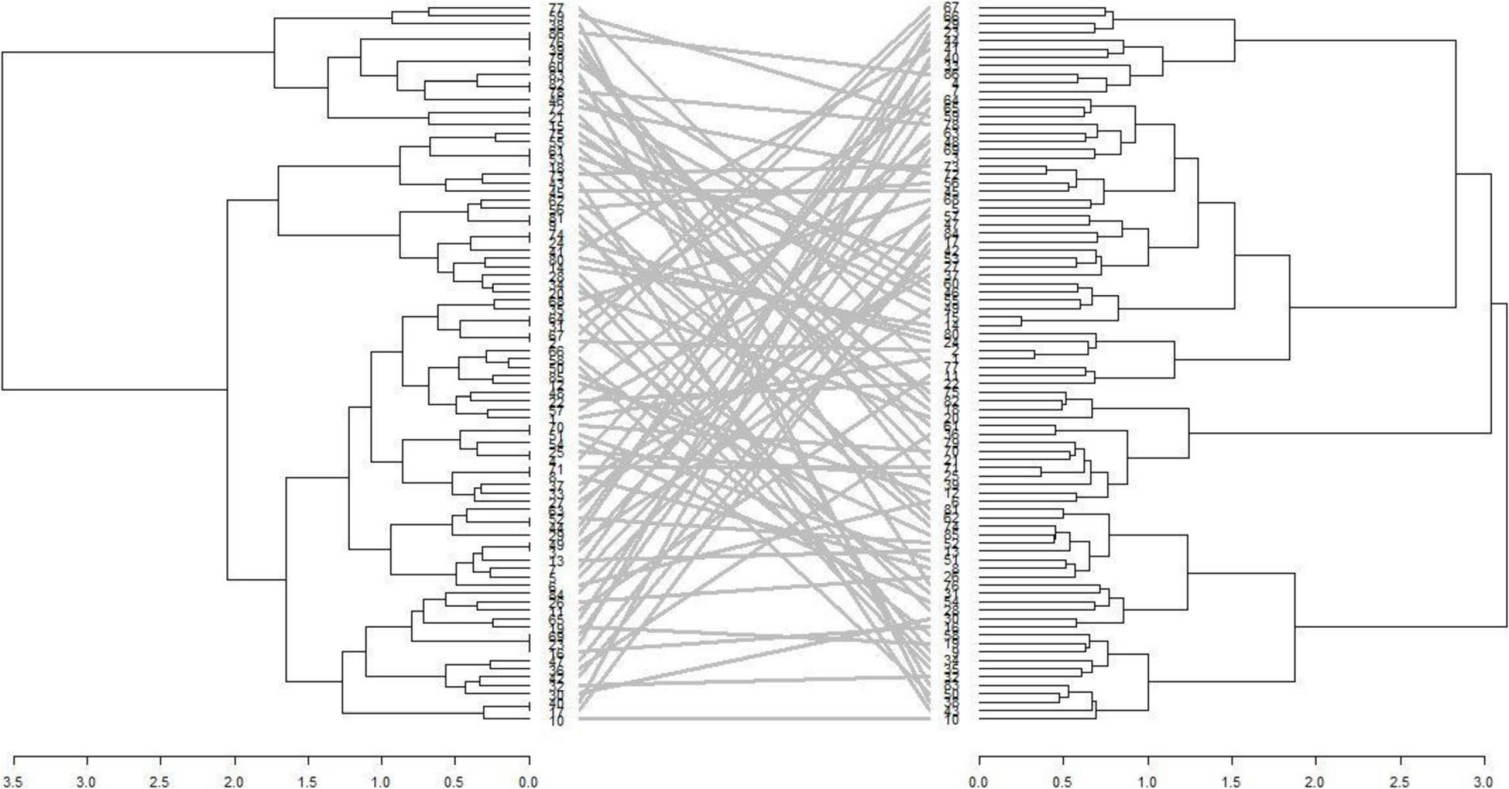
Tanglegram showing comparison of phenotypic and genotypic dendrograms

The dendrograms for phenotypic and genotypic data each revealed three heterogeneous clusters among the genotypes. The phenotypic dendrograms showed that the first cluster comprised of genotypes from all sources, while the second cluster comprised of elite lines from Malawi, University of Zambia and the National Gene Bank and the last cluster contained accessions from the National Gene Bank, Malawi and the University of Zambia (Fig. 3). The largest cluster in the genotypic dendrogram contained genotypes from the National Gene Bank, while the second largest consisted of mostly genotypes from Malawi (Fig. 4). The joint matrix revealed three similarly sized clusters among the genotypes (Fig. 5). The phenotypic and genotypic dendrograms were compared using the tanglegram and only a few genotypes (about 10%) maintained their positions (Fig. 6). The tanglegram comparison highlighted that both the positions and groupings of the genotypes were not consistent across the phenotypic and genotypic dendrograms. A relatively few genotypes maintained their position, i.e., about 30% maintained their groupings. Furthermore, the phenotype and genotype dissimilarity matrices exhibited a very low correlation (*r* = − 0.03) when subjected to the Mantel test (Additional files 2 and 4). In contrast, the genotype and phenotype dissimilarity matrices each exhibited strong correlations of *r* = 0.12, and *r* = 0.99 with the joint matrix, respectively (Additional files 5 and 6).

## Discussion

### Genotypic variation and performance of test genotypes for key qualitative and quantitative traits

The present study evaluated the genetic diversity present among 100 diverse genotypes of cowpea germplasm collections from southern Africa using qualitative and quantitative phenotypic traits in two locations in Zambia. Further, high density SNP markers were used as a preliminary step to identify suitable and complementary parental lines for breeding. There were significant genotype × location interaction (Table 9) effect signifying that the tested germplasm were genetically diverse for selection and cultivar development targeting the test locations. Also, the interaction effect shows that the genotypes responded differently in the test environments which can facilitate identification of cowpea lines with specific or broad adaptation. Specific and broad adaptation have been identified and exploited in the Brazilian cowpea breeding programs based on genotype × location interaction analysis [21]. The interaction effect suggests that the test environments influence genotypic performance, which may confound genotype selection efforts by reducing the correlation between genotype and phenotypic expression [22].

**Table 9:** Qualitative and quantitative traits of cowpea assessed during the study

No	Trait	Abbreviation	Trait description
**Qualitative traits**			
1	Flower colour	FLC	Flower colour intensity: violet-1, yellow − 2, white- 3
2	Leaf green colour	LGC	Colour intensity: light-1, medium − 2, dark- 3
3	Growth pattern	GTH	Type 1 - determinant, type 2- indeterminate, type 3- creeping
4	Pod colour	PDC	Pod colour intensity; light green-1, deep green − 2, purple- 3
5	Seed coat colour	STC	Primary colour intensity of the seed coat; reddish- brown −1, white − 2, purplish- brown − 3, brown - 4, black - 5,
6	Leaf size	LFS	Size of the most tip leaf; small − 1, medium −2, big −3
**Quantitative traits**			
1	Days to 50% flowering	DTF	The number of days from sowing until 50% of the plants in a plot have visible flowers
2	Days to 90% maturity	DTM	Days from date of sowing to the date when 90% of pods in a plot turn yellowish brown
3	Number of pods per plant	NPP	Mean number of mature pods from 10 randomly selected and tagged plants in a plot
4	Pod length	PDL	Mean length of 10 mature pods from randomly selected and tagged plants
5	Number of seeds per pod	NSP	Mean weight of seed from mature pods of 10 randomly selected and tagged plants
6	Seed size	SDS	Mean length of 10 randomly selected seed measured in millimetres
7	Hundred seed weight	HSW	Weight of one hundred randomly selected seeds of a genotype measured in grams
8	Grain yield	GYD	The average grain yield per plot and converted into kg ha^−1^ using the formula given above.

In the present study, the assessed quantitative traits were affected by genotype × location interaction effect. Hence, there is intrinsic genetic variation influenced by the test locations necessitating multi environment evaluation for selection. Differential genotype response to environmental conditions during germplasm evaluation is attributable to the differences in genetic constitution among test genotypes and micro-environmental conditions [23]. In this study, the SCCI (mean yield of 832 kg ha^− 1^) site is high yielding environment compared with the GART (mean yield of 764 kg ha^− 1^) site probably due to the prevailing favourable environmental conditions such as better soil fertility and higher moisture levels in the former. Genotype phenology and biomass production exhibit environmental plasticity due to variable soil and climatic factors [24, 25]. In the present study, some genotypes were high grain yielders (e.g. CP411 with 2197 kg ha^− 1^) and others were low yielders (e.g. ZM6680 with 87 kg ha^− 1^. Quantitative traits are under the influence of polygenes. Hence, it is pertinent for genotype selections in multiple test environments to minimise environmental variance and to enhance selection gains [26, 27]. Genotypes such as MS1–8–1-4, Msandile, BBXSC13, CP411, CP421, CP654, CP3413 and Bubebe that exhibited early to medium maturity are ideal candidates for drought tolerance breeding to offset the incessant droughts experienced in southern Africa. Early maturity is associated with drought escape [28] [8]. reported that farmers in southern Africa prefer cultivars with a short flowering period and maturity, valuable traits to evade the “hunger period”. Highest number of seeds per pod (e.g. expressed by genotypes CP421 and Bubebe) is one of the factor affecting genotype responses based on their efficiency in growth resource utilisation and allocation. This could also be contributed to increased length of the pods by test genotypes [29]. Seed weight is directly associated with seed size and it is recommended to be used as an indirect selection criterion to maximise grain yield response in cowpea [10] [8]. reported a high yield potential of cowpea genotypes that can reach up to 3 t/ha. The yield level recorded in the present study by the landraces was relatively less. This could be the low yield potential of landraces grown by most farmers in SSA. In the region, landraces are continuously cultivated because they possess farmer-preferred quality traits and their ability to adapt under variable stress conditions due to their genetic diversity and plasticity [30, 31].

In the present study qualitative traits such as seed coat, pod and leaf colour were more important traits for selection. These traits affect the market value of cowpea in Africa given that farmer and consumer preference are based on these attributes. Seed coat colour is often associated with processing quality (e.g. cooking time) and farmers deliberately select varieties that have shorter cooking time [32]. The inheritance of seed coat colour is governed by few major genes that will enhance selection progress during cultivar development [33]. In this study, the genotypes Bubebe and Msandile, with predominantly light-green leaves exhibited determinate growth habit in comparison with BBXSC13 and Chimponongo that had dark green leaves and creeping growth habit [34]. reported that cowpea cultivars with a determinate growth type were more drought tolerant compared to the indeterminate types [35]. reported that indeterminate varieties of cowpea attained higher productivity due to their prolonged maturity and photosynthesis efficiency. Therefore, in order to promote sustainable production and productivity and enhanced adoption of improved cowpea cultivars, breeding programs should incorporate farmer- and market- preferred attributes in the newly developed cultivars.

### Variance, heritability and genetic advance

In this study, the heritability estimate for grain yield was high (64%), suggesting that the grain yield achieved by the accessions was highly repeatable ensuring genetic improvement through selection. The high heritability value for grain yield corroborates with the findings of [2] but lower than a heritability value of 97% reported by [29]. Genetic advance is directly related with yield gains achievable via selection. High estimates of genetic advance (e.g. for HSW and SDS) and high heritability indicate that selection would result in foreseeable genetic improvement [36, 37]. The large discrepancies values of PCV compared to GCV in this study, suggests that trait expression was also influenced by environment factors in addition to genetic effects, which was also confirmed by the significant location main effects in the ANOVA (Table 2).

### Associations of quantitative traits

The relationships among yield and yield components are critical in devising a selection strategy. Selection of one trait may amplify or negatively affect performance in the other traits. The high contribution and strong association of PDL, NSP, GYD, and NPP to PC1 as well as DTF and DTM with the PC2 indicated that these traits were highly discriminatory explaining the variation among the genotypes [38, 39]. found that traits such as NPP and GYD in cowpea were associated with PC1 showing the importance of agronomic traits in cowpea evaluation corroborating with the findings of the present study. The genotype-trait biplot enables visual and simultaneous selection of genotypes for multiple traits. There was strong correlations between PDL, NPP, NSP and grain yield indicating their positive impact on genotype performance. Previous reports identified these traits being important yield-influencing attributes [29, 39]. Entries such as E10 (CP 411), E71 (LT16–7–2-5), E13 (CP421) and E20 (CP645) scored greater grain yield response and yield-influencing traits suggesting their utility in variety improvement for yield gains and breeding population development. Entries such as E10 (CP411), E20 (CP645), E13 (CP421) and E58 (Sundan1) are selected with desirable NSP, GYD, PDL and DTF, respectively.

### Population structure and genetic parameters

Genetic analysis using SNP markers delineated the test populations in to four genetic groups. This demarcation was irrespective of source of collection, suggesting that geographical sources of collection are not the sole factor for classification of cowpea genotypes. The germplasm exhibited high heterozygosity of 0.30, which showed that alternative alleles were represented in this population. This could be attributed to genetic exchange between and among cowpea breeding programs in sub-Sahara Africa, particularly in southern Africa. Heterozygosity estimates were highest for mutant lines, followed by elite lines and landraces. This suggests that mutant lines exhibited more variability possibly due to continuous gene segregation and other chromosomal aberrations. Landrace varieties are not subjected to artificial selection by breeders. Hence, landraces of self-pollinating crops including cowpea consists of heterogeneous populations due to genetic variation until complete homozygosity is reached through selection [40]. Mutant and elite lines are the result of several cycles of deliberate selection and inbreeding with expectedly high level of homozygosity at multiple loci. The present mutant lines were grouped together with the mother stocks or parental lines. This shows that the mutation events were minor and the genetic background largely remained the same for most of the assessed traits in the mutants lines compared with the elite parental lines. Malawi and Zambia are in close geographical proximity hence germplasm exchange between the two countries cannot be ruled out. Trait preference for the farmers and the market in the region may not be significantly different leading to the overlap of cowpea genetic resources in these agro-ecologies. This has partly disallowed the population structure analysis without distinguishing the genotypes based on geographical sources agreeing to the report of [18]. Exchange of genetic resources is key for plant-breeding research and cultivar development, which are dependent on wider genetic bases [10].

The PIC and GD values were essential to assess genetic diversity within the whole population, and sub-populations for identification selection of divergent parental lines useful for breeding. The moderate PIC and GD values exhibited by the whole population could be attributed to the inherent nature of self-pollination in cowpea, which limits genetic diversity. Self-pollinating species often lack genetic diversity due to higher levels of homozygosity at multiple loci. The within-population diversity in self-pollinating species is relatively low but varies among populations [41, 42]. In contrast, mutants lines exhibited higher Ho, PIC and GD values compared to the elite lines or landraces. Naturally, landraces of self-pollinating species would be expected to exhibit higher levels of homozygosity compared to mutant and elite lines that were subjected to deliberate mutagenesis and crossing during breeding. On the hand, mutant lines exhibited higher heterozygosity and PIC values than elite lines, which could be due induced random mutations. Mutation breeding is a well-established technique to widen genetic diversity [43]. However, the mutant and elite lines displayed difference in growth pattern. This may be attributed to genetic differences in plant architecture in terms of growth habit and maturity period, among others. For example, the elite line Bubebe and the mutant line MS1–8–1-4, have short maturity period and determinate growth type. Hence, inclusion of landraces such as Chimponongo and Kapita with late maturity and creeping growth habit would be recommended to increase genetic variation and to enhance genetic gain through selection. This is consistent with the findings of other cowpea researches who indicated that architecture of the crop results in genetic diversification [9]. The mean Fst values recorded in the present study showed low genetic differentiation among the test populations (max Fst = − 0.012), which could be attributed to the self-pollination nature of cowpea. This shows that the sub-groups account for a small proportion of the total variance exhibited by the whole population and selection would be more efficient between genotypes rather than between sub-groups. However, the negative Fis values indicate that individuals accounted for a large proportion of the total variance due to the presence of heterozygotes that could be useful in future selection programs. This could due to high heterozygosity values estimated for the whole germplasm underpinned by mutants and elite lines that have undergone artificial selection, and landraces that may be exhibiting segregation. Individual selection will enhance genetic advance compared to selection within a sub-group.

The phenotypic and genotypic distance matrices exhibited low and non-significant correlation, which confirm that phenotypic and genotypic matrices were independent but complementary. The discordance between phenotypic and genotypic matrices is partly attributed to the significant environmental effect on the variable expression of the assessed phenotypic traits. Phenotypic performance is the result of the genotype, environment and genotype-by-environment interaction effects [44]. In addition, the inconsistences between genotypic and phenotypic matrices could be due to the fact that genotyping may be able to capture subtle genetic mutations across the whole genome that may not be expressed phenotypically. Other studies reported inconsistences between phenotypic and genotypic matrices in common beans (*Phaseolus* spp) [45]), yam (*Dioscorea* spp) [46] and *Brassica* spp. [46]. Due to the expected discord between genotypic and phenotypic matrices, the use of a joint matrix derived from combined phenotypic and genotypic matrices has been recommended to increase precision [47, 48]. The strong correlations exhibited by phenotypic and genotypic matrices each with the joint matrix show that each were derivative of the joint matrix and can be used for increased precision as they do not overlap. An increase of more than 150% in precision was reported while calculating dissimilarity distances using a joint matrix compared to the phenotypic matrix [49].

## Conclusions

Phenotypic analysis using qualitative and quantitative traits and genotyping using high density SNP markers revealed the presence of significant variation among 100 cowpea germplasm collections of southern Africa. Trait association analysis revealed significant correlation between NPP, NSP, PDL and GYD that could allow direct selection to improve grain yield. The SNP markers used in the study were able to deduce genetic variation among the tested cowpea populations. The largest proportion of variation was attributable to individual genotype differences that is essential for improving grain yield by crossing lines from different divergent populations. Test genotypes were classified in to four genetic groups irrespective of source of collection allowing selection for subsequent cross combinations to develop breeding populations for cultivar development. Genotypes Bubebe, CP411, CP421, CP645, Chimponogo and MS1–8–1-4 were identified being the most genetically divergent and high yielding making them ideal parental lines for breeding. This study provided a baseline genetic profile and identified promising cowpea genetic resources for effective breeding and systematic conservation.

### Plant materials

The study evaluated 90 cowpea genotypes acquired from different sources (Supplementary Table 1). The germplasm included 61 elites lines from Malawi, International Institute for Tropical Agriculture (IITA), Nigeria, The National Gene Bank of Zambia and the University of Zambia, 15 landraces collected from smallholder farmers in Zambia and 14 mutant lines, which were derived from three parental lines; Lutembwe, Bubebe and Msandile. The accessions from IITA and the released cultivars were used as standard checks.

### Phenotyping

#### Description of the study sites

The 100 genotypes were field evaluated during the 2017/2018 main crop season at the following two sites: the Seed Control and Certification Institute (SCCI) in Chilanga and Golden Valley Agricultural Research Trust (GART) in Chisamba/Zambia. The SCCI site is situated at a latitude of 15^o^ 32′S and a longitude of 28^o^11’E with an altitude of 1206 m above sea level. The total mean annual rainfall at the SSCI site is 1092 mm, while the mean daily minimum and maximum temperatures were 12 °C and 26 °C, respectively. The GART site is situated at a latitude of 14^o^ 96′S and a longitude of 28^o^10’E and an altitude of 1103 m above sea level. The GART site receives a total mean annual rainfall of 884 mm with mean daily minimum and maximum temperatures of 10 °C and 30 °C, respectively. The soils at both sites are classified as Haplustalf clays with pH of 5.8 and 5.2 at SCCI and GART, respectively [50].

#### Trial design, field planting and management, and data collection

The experiments were laid out in a 10 × 10 alpha lattice design with two replications. Each genotype was sown in a plot with two rows of 5 m long. The plot area was 3.75m^2^. The inter-row and intra-row spacings were 75 and 45 cm, respectively. Two seeds were sown per station at a depth of 2 cm and later thinned to one plant two weeks after emergence. Basal fertiliser (N: P: K), containing 20% nitrogen, 10% phosphorus and 20% potassium, was applied at a rate of 200 kg ha^− 1^ prior to planting. All other agronomic practices for cowpea production were followed as recommended for Zambia [50]. The crops were grown under rain-fed conditions and both sites received an annual rainfall of 850 mm during the study.

### Data collection

Data was collected from six qualitative and eight quantitative traits following the descriptors of the [15, 51]. The list of traits and details of data collection and units are provided in Table 2. Grain yield was determined in kg ha^− 1^ based on the following formula:

$\frac{plot\ weight}{plot\ area}\ \mathrm{x}\ \frac{100-14}{100- mc}\kern0.5em \times 10,000$ where; mc is moisture content measured at harvesting, 14% is standard constant moisture content for legumes [51] and 10,000 is a conversion factor for a hectare.

### Data analysis

The frequency of test genotypes displaying the assessed qualitative traits were summarised and statistical significant tests conducted using the cross tabulation procedure with the Statistical Package for the Social Sciences (SPSS) version 24 [52]. The quantitative data was subjected to analysis of variance (ANOVA) using the alpha-lattice procedure in GenStat® version 18 [53]. A combined analysis of variance was conducted after detecting significant differences among tested genotypes in each location. The following linear model was used for the combined analysis of variance: *β*ijk = μ + Gi + Ej + Gi ∗ Ej + Ei (rk)(b) + *ε*ijk, where; *β*ijk = observed response; *μ* = grand mean Gi = the effect of i^th^ genotype; Ej = the effect of j^th^ location, Gi ∗ Ej = the genotype x location interaction effect; Ej(rk)(b) = error associated with k^th^ replication in blocks in the j^th^ location and *ε*ijk = experimental error. The blocks within replications were considered as random factor, while genotypes and locations were fixed factors. Trait means of test genotypes were separated using the Fischers Unprotected LSD at *p* ≤ 0.05 significance level. Genotypic, genotype by location interaction and phenotypic variances were computed from the excepted mean squares of the analysis of variance as follows; ${\sigma}^2g=\frac{\left( msg- mse\right)}{lr};{\sigma}^2 gl=\frac{\left( msg l- mse\right)}{r};{\sigma}^2\mathrm{p}={\sigma}^2g+{\sigma}^2\mathrm{e}+{\sigma}^2 gl$, where; *σ*^2^*g* = genotypic variance, *σ*^2^*gl* = genotype by location interaction variance, *σ*^2^ : *p* = phenotypic variance, msg = mean square of genotype, mse = mean square of error, l = number of location and *r* = number of replication. Variances below zero were adjusted to zero according to [54]. Heritability in broad sense (H^2^) was computed according to [55]; ${\mathrm{H}}^2=\frac{\upsigma^2g}{\upsigma^2p}x\ 100$ where; *σ*^2^*g* is genotypic variance and *σ*^2^*p* is phenotypic variance. Heritability was categorized as low (0–0.30), moderate (0.30–0.60) and high (> 0.60) following [56]. A covariance analysis was performed to calculate coefficient of variations. The genotypic coefficient of variation (GCV) and phenotypic coefficient of variation (PCV) expressed in percent were computed as described by [57] as follows: $\mathrm{GCV}=\left(\frac{\sqrt{\upsigma^2}g}{\frac{\kern0.75em }{\upchi}}\right)\times 100;\mathrm{PCV}=\left(\frac{\sqrt{\upsigma^2}p}{\frac{\kern0.75em }{\upchi}}\right)\times 100$, were *σ*^2^*g* = genotypic variance, *σ*^2^p = phenotypic variance, $\frac{\kern0.75em }{\upchi}$ = grand mean. Genetic advance was calculated following [58] as follows: GA = (*k*) (σp) (*h*^2^), where, GA = Genetic advance; *k =* selection differential at 5% selection intensity; σp = phenotypic standard deviation; *h*^*2*^ = broad sense heritability; Genetic advance as a per cent of mean (GAM) was computed following [59]: $\mathrm{GAM}=\left[\frac{\mathrm{GA}}{\frac{\kern0.75em }{\upchi}}\right]\times 100$, where, GA = (%) = Genetic advance as a per cent of mean; GA = Genetic advance; $\frac{\kern0.75em }{\upchi}$ = Grand mean. Genetic advance as a per cent of mean was classified and rated based on the scales given by [60] as low (< 10%), moderate (10–20%) and high (> 20%).

The magnitude of traits relationship was determined using Pearson’s correlation coefficients (*r*) using the SPSS version 24 [52]. Principal component analysis (PCA) was performed using the same software to examine the number principal components and trait associations. The principal components (PCs) with Eigen-values ≥1.0 were considered to explain the variation in phenotypic traits among the genotypes. PCA biplots were constructed in GenStat [53] to depict the relationships among the studied genotypes and traits.

### Genotyping

#### DNA isolation and genotyping

Ten seeds of each cowpea genotype were planted in a plastic pot. The seedlings were allowed to grow to the three-leaf stage before fresh leaves were harvested for DNA extraction. Leaves were sampled from each genotype for DNA extraction. Fifty milligrams of total genomic DNA was extracted from the well-developed trifoliate leaves with the NucleoSpin plant II kit (Macherrey- Nagel, Duren, Germany) using the Lysis Buffer 1 (based on the CTAB method) according to the manufacturer’s procedures. The DNA concentration of each sample was measured using a NanoDrop 1000 (Invitrogen, California, USA). For verifying DNA integrity, 2 μL of DNA were subjected to gel electrophoresis on 1.0% (w/v) agarose gel, stained with ethidium bromide. Subsequently, 40 μL of a 50 ng/μL DNA of each sample were genotyped with Illumina Cowpea iSelect Consortium Array using Diversity Arrays Technology (DArT) markers. In total, 94 cowpea genotypes were genotyped by the genotyping by sequencing (GBS) technology as described by [61] with 20,000 DArT markers. The markers were integrated into a linkage map by inferring marker order position from the consensus Dart map. Genotyping of the materials was carried out at the Biosciences eastern and central Africa- International Livestock Research Institute (BecA- ILRI) in Kenya.

### Data analyses

#### SNP filtering

For quality control, DArTseq SNP derived markers were filtered to remove bad SNPs and genotypes using the software’s PLINK 1.9 in MS window and R statistical package. Markers and genotypes with > 20% missing data were eliminated. Rare SNPs with < 5% minor allele frequencies were also pruned from the data. Only 14,116 informative SNP markers and 90 genotypes were used for analysis after removal of 4240 SNPs and four genotypes. The four genotypes, CP1, CP2, CP479, and CP2223 were removed due to extreme heterozygosity (< 90%), duplication or high levels of missing data (> 20%). The chromosomal coverage of the 14,116 SNPs was presented in Supplementary Fig. 3.

### Population structure and genetic diversity analysis

The Bayesian clustering method was used for infering the population structure of the germplasm using the STRUCTURE version 2.3 software [62]. The STRUCTURE settings were set at a burn-in period of 5000 and 5000 Monte Carlo Markov Chain (MCMC) iterations with an admixture model to deduce the number of clusters using K values between 1 and 10. The best K- value for estimating a suitable population size was identified by the Evanno method in the online based Structure Harvester program [63]. After estimating the best K, a new run using a burn-in period of 100,000 and 100, 000 MCMC was performed to assign accessions to sub-populations. The accessions with a membership probability lower than 0.80 of a sub-population were assigned to an admixture group. The genetic structure was further assessed using a Neighbour Joining tree method [64]. Principal component analysis was conducted in TASSEL v.5 [65] using the 14,116 SNPs and plotted using TIBCO spotfire 6.5.0. A dendogram was generated using hierarchical clustering method [66]. The expected heterozygosity (He) and polymorphism information content (PIC) were calculated using [67].

#### Joint analysis of phenotypic and genotypic data

A joint analysis based on a combination of phenotypic and genotypic dissimilarity matrices was conducted. A Gower’s distance matrix was generated from the phenotypic data while the genotype dissimilarity matrix was computed based on Jaccard’s coefficient. The correlations among the phenotypic, genotypic and joint dissimilarity matrices were compared using the Mantel’s test with 999 permutations [68] using the package “clusters” and the Daisy procedure [69]. The dissimilarity matrices were used to generate hierarchical clusters in the package “cluster” in R software [69] and compared using the tanglegram function in “dendextend” package in R software [70].

## Supplementary information

**Additional file 1.** Statistical tests and distribution of 100 cowpea germplasm collections based on qualitative traits.

**Additional file 2. **Correlations among phenotypic, genotypic and joint matrices based on Mantel test with 999 permutations showing the correlations (above diagonal) and *p*-values (below diagonal).

**Additional file 3.** List, source, and description of 90 cowpea genotypes used in the study.

**Additional file 4.** The Mantel test between phenotypic and genotypic matrices.

**Additional file 5.** The Mantel test between phenotypic and joint matrices.

**Additional file 6.** The Mantel test between genotypic and joint matrices.

**Additional file 7.** Chromosomal distribution of the 14,166 SNPs used to evaluate 90 cowpea genotypes.

## Data Availability

All data generated or analysed during this study are included in this published article and its supplementary information files.
