# Supplementary material for: Assessing the genetic diversity of cowpea [Vigna unguiculata (L.) Walp.] germplasm collections using phenotypic traits and SNP markers
Source: BMC Genet. 2020 Sep 18;21:110. doi: 10.1186/s12863-020-00914-7 (PMC7501654; doi:10.1186/s12863-020-00914-7)
Supplement: Supplementary file 1 — Additional file 1. Statistical tests and distribution of 100 cowpea germplasm collections based on qualitative traits. [file 12863_2020_914_MOESM1_ESM.docx]

Additional file 1. Statistical tests and distribution of 100 cowpea germplasm collections based on qualitative traits

| **Trait** | **Description** | **Frequency (%)** | **Degrees of freedom** | **Chi-square** | **P-value** | **Genotypes** |
| --- | --- | --- | --- | --- | --- | --- |
| Growth habit | Determinate | 43.0 | 198 | 469.19 | 0.00 | BB3-9-7-5, BB8-1-5-2, BB10-4-2-5, BB14-16-2-2, BBXSC13, BBSC12, Bubebe, CP1, CP2, CP6, CP11, CP414, CP418, CP732, CP753, CP2232, CP2980, CP3067, CP3422, CP3423, CP3425, Geneb, IT82-16, IT82-16E, Kapita, L10xL7, Local chipata, LT3-8-4-1, LT4-2-4-1, LT11-3-3-12, LT11-3-3-13, LT11-5-2-2, Lutechipata, Lutembwe, MS1-8-1-4,Sundan1, ZM2960, ZM2966, ZM3064, ZM3716, ZM4588, ZM4706, ZM6680. |
|  | Indeterminate | 39.0 |  |  |  | CP4,CP12, CP102,CP305, CP411, CP421, CP426,CP479,CP601,CP645,CP1769, CP2231,CP2863,CP3420,CP2XSC103, Chawa, Kapita black, Kapita north,L4XL3, L8XL9, Local kapita, LT16-7-2-5, Lute, Makulu, Mount, Namuseba, ZM308, ZM471, ZM1790, ZM2095, ZM2938, ZM2943, ZM2954, ZM2969, ZM2999, ZM3000, ZM3070, ZM4710, ZM5419. |
|  | Creeping | 18.0 |  |  |  | Bgene, CP399, CP436, CP570, CP633, CP698, CP2223, CP3413, Chiko, Chimponongo, LT4-2-4-14, Msandile, Mtilizi, Muz, ZM2081, ZM2108, ZM2939, ZM3003 |
| Leaf size | Small | 35.0 | 198 | 404.31 | 0.00 | BBXSC13, BB8-1-5-2, CP6, CP12, CP421, CP645, CP698, CP1769, CP2232, CP2863, CP3422, Chiko, IT82-16E, L8XL9, LT4-2-4-14, LT4-2-4-14, LT11-3-3-12, Lutembwe, Lutechipata, MS1-8-1-4, Sundan1, ZM2095, ZM2108, ZM2939, ZM2943, ZM2954, ZM2966, ZM3003, ZM3064, ZM3070, ZM3716, ZM4588, ZM4706, ZM5419. |
|  | Medium | 26.0 |  |  |  | BB10-4-2-5, BB14-16-2-2, BBSC12, Bgene, Bubebe, CP2, CP4, CP11, CP414, CP418, CP436, CP753, CP2231, Chawa, Geneb, L10Xl7, Local Chipata, LT3-8-4-1, LT11-3-3-13, LT11-5-2-2, Namuseba,Makulu,ZM1790, ZM2960, ZM2969, ZM5419. |
|  | Big | 39.0 |  |  |  | BB3-9-7-5, CP1,CP102, CP399, CP411, CP426, CP479, CP570, CP601, CP633, CP732, CP2223, CP2980, CP3067, CP3413, CP3420, CP3423, CP3425, CPXSC103, Chimponongo, IT82-16, Kapita, Kapita black, Kapita north, L4XL3, Local kapita, LT16-7-2-5, Lute, Mount, Msandile, Mtilizi, Muz, ZM308, ZM471, ZM2081, ZM2938, ZM3000, ZM6680. |
| Flower colour | White | 1.0 | 198 | 387.81 | 0.00 | ZM6680. |
|  | Yellow | 4.0 |  |  |  | CP11, Namuseba, MS1-8-1-4, ZM2095. |
|  | Violet | 95.0 |  |  |  | BB3-9-7-5, BB8-1-5-2, BB10-4-2-5, BB14-16-2-2, BBXSC13, BBSC12, Bgene, Bubebe, CP1, CP4, CP2, CP6, CP11, CP12, CP102, CP305, CP399, CP411, CP414, CP418, CP421, CP426, CP436, CP479,CP601, CP633, CP645, CP698, CP732, CP753, CP421, CP426, CP479, CP570, CP601, CP645, CP1769, CP2223 CP2231, CP2232, CP2863, CP2980, CP3067, CP3413, CP3420, CP3422, CP3423, CP3425, CP2XSC103, Chawa, Chiko, Chimponongo, Geneb, IT82-16, IT82-16E, Kapita, L10xL7, Local Chipata, LT3-8-4-1, LT4-2-4-14, LT4-2-4-1, LT11-3-3-12, LT11-3-3-13, LT11-5-2-2, Kapita black, Kapita north, L4XL3, L8XL9, Local kapita, LT16-7-2-5, Lute, Lutechipata, Lutembwe, Makulu, Mount, MS1-8-1-4, Msandile, Mtilizi, Muz, Namuseba, Sundan1, ZM300, ZM308, ZM471, ZM1790, ZM2081, ZM2095, ZM2108, ZM2938, ZM2939, ZM2943, ZM2954 ZM2960, ZM2966, ZM2969, ZM2999, ZM3000 ZM3064, ZM3070, ZM3716, ZM4588, ZM4706, ZM4710, ZM5419, ZM6680. |

Additional file 1 …. Continued

| **Trait** | **Description** | **Frequency (%)** | **Degrees of freedom** | **Chi-square** | **P-value** | **Genotypes** |
| --- | --- | --- | --- | --- | --- | --- |
| Pod colour | Light green | 30.0 | 198 | 445.12 | 0.00 | BB10-4-2-5, BBSC12, BBXSC13, BB3-9-7-5,CP2,CP11, CP414, CP418,CP753, CP2231, CP2232, CP23, CP3425, IT82-16E, Kapita black, L8XL9, LT3-8-4-1, LT4-2-4-1, LT11-3-3-13, LT11-5-2-2, MS1-8-1-4, Namuseba, ZM2095, ZM2938, ZM2999, ZM3716, ZM4706, ZM4710, ZM5419. |
|  | Dark green | 52.0 |  |  |  | BB8-1-5-2, BB14-16-2-2, Bgene, CP1, CP4, CP6, CP12, CP305, CP426, CP436, CP479,CP570, CP601, CP645, CP732, CP1769, CP2863, CP2980, CP3413, CP2XSC103, Chawa, Chimponongo, Geneb, IT82-16,Kapita, Kapita north, Local chipata, Local kapita, LT4-2-4-14, LT11-3-3-12, Lutechipata, Lutembwe, Msandile, Mtilizi, Muz, Sundan1, ZM308, ZM471, ZM1790, ZM2081, ZM2108, ZM2939, ZM2954, ZM2960, ZM2969, ZM3000, ZM3003, ZM3070, ZM4588, ZM6688. |
|  | Purple | 18.0 |  |  |  | CP102, CP399, CP411, CP421, CP633, CP698, CP2223, CP3067, CP3420, CP3422, Chiko, L4Xl3, LT16-7-2-5, Lute, Makulu, Mount, ZM2943, ZM3064. |
| Leaf colour intensity | Light green | 26.0 | 198 | 588.10 | 0.00 | BB10-4-2-5, BB14-16-2-2, BBSC12, Bgene, Bubebe, CP2, CP4, CP11, CP414, CP418, CP436, CP753, CP2231, Chawa, Geneb, L10Xl7, Local Chipata, LT3-8-4-1, LT11-3-3-13, LT11-5-2-2, Namuseba,Makulu,ZM1790, ZM2960, ZM2969, ZM5419. |
|  | Medium green | 35.0 |  |  |  | BBXSC13, BB8-1-5-2, CP6, CP12, CP421, CP645, CP698, CP1769, CP2232, CP2863, CP3422, Chiko, IT82-16E, L8XL9, LT4-2-4-14, LT4-2-4-14, LT11-3-3-12, Lutembwe, Lutechipata, MS1-8-1-4, Sundan1, ZM2095, ZM2108, ZM2939, ZM2943, ZM2954, ZM2966, ZM3003, ZM3064, ZM3070, ZM3716, ZM4588, ZM4706, ZM5419. |
|  | Dark green | 39.0 |  |  |  | BB3-9-7-5, CP1,CP102, CP399, CP411, CP426, CP479, CP570, CP601, CP633, CP732, CP2223, CP2980, CP3067, CP3413, CP3420, CP3423, CP3425, CPXSC103, Chimponongo, IT82-16, Kapita, Kapita black, Kapita north, L4XL3, Local kapita, LT16-7-2-5, Lute, Mount, Msandile, Mtilizi, Muz, ZM308, ZM471, ZM2081, ZM2938, ZM3000, ZM6680. |
| Seed coat colour | Red- brown | 8. 0 | 396 | 557.10 | 0.82 | BBXSC13, CP2, CP570, CP3420, L4XL3, Msandile, MS1-8-1-4, ZM2966. |
|  | White | 10.0 |  |  |  | BB8-1-5-2, BB8-1-5-2, BB10-4-2-5, BB14-16-2-2, Bubebe, CP2XSC103, L8XL9, L10XL7, Nanuseba, ZM3716. |
|  | Purple- brown | 12.0 |  |  |  | Bgene, CP102, CP418, CP3435, Chiko, Geneb, Kapita north, LT11-3-3-13, Lute, Mtilizi, Mount, ZM2969 |
|  | Brown | 49.0 |  |  |  | BBSC12, CP1,CP6,CP11,CP305,CP399, CP411, CP414,CP421, CP436, CP466, CP633, CP645, CP668, CP753, CP1769, CP2223, CP2232, CP2863, CP3067, CP3413, CP3422, Chawa, Kapita, Local kapita,LT4-2-4-1, LT4-2-4-14, LT11-3-3-12, LT11-5-2-2, LT16-7-2-5, Lutechipata, Lutembwe, Makulu, Muz, Sundan1, ZM308, ZM471, ZM2095, ZM2938, ZM2943, ZM2954, ZM2960, ZM2999, ZM3064, ZM3067, ZM4706, ZM4710, ZM5419, ZM6680 |
|  | Black | 21.0 |  |  |  | CP4, CP12, CP479, CP601, CP732, CP2231, CP2980, CP3423, Chimponongo, IT82-16, IT82-16E, Kapita black, Local Chipata, ZM1790, ZM2081, ZM2108, ZM2929, ZM3000, ZM3003, ZM3070, ZM4588. |
