# Supplementary material for: Assessing the genetic diversity of cowpea [Vigna unguiculata (L.) Walp.] germplasm collections using phenotypic traits and SNP markers
Source: BMC Genet. 2020 Sep 18;21:110. doi: 10.1186/s12863-020-00914-7 (PMC7501654; doi:10.1186/s12863-020-00914-7)
Supplement: Supplementary file 2 — Additional file 2. Correlations among phenotypic, genotypic and joint matrices based on Mantel test with 999 permutations showing the correlations (above diagonal) and p-values (below diagonal). [file 12863_2020_914_MOESM2_ESM.docx]

Additional file 2. Correlations among phenotypic, genotypic and joint matrices based on Mantel test with 999 permutations showing the correlations (above diagonal) and p-values (below diagonal)

| Matrix | Phenotypic | Genotypic | Joint |
| --- | --- | --- | --- |
| Phenotypic | - | -0.025 | -0.125 |
| Genotypic | 0.69 | - | 0.99 |
| Joint | 0.7 | 0.00 | - |
