# Supplementary material for: Assessing the genetic diversity of cowpea [Vigna unguiculata (L.) Walp.] germplasm collections using phenotypic traits and SNP markers
Source: BMC Genet. 2020 Sep 18;21:110. doi: 10.1186/s12863-020-00914-7 (PMC7501654; doi:10.1186/s12863-020-00914-7)
Supplement: Supplementary file 3 — Additional file 3. List, source, and description of 90 cowpea genotypes used in the study. [file 12863_2020_914_MOESM3_ESM.docx]

Additional file 3. List, source, and description of 90 cowpea genotypes used in the study

| No. | Genotype | Source | Description | No. | Genotype | Source | Description |
| --- | --- | --- | --- | --- | --- | --- | --- |
| 1 | CP 1 | NGB | Elite line | 46 | ZM 2081 | NGB | Elite line |
| 2 | CP 2 | NGB | Elite line | 47 | ZM 2095 | NGB | Elite line |
| 3 | CP 4 | NGB | Elite line | 48 | ZM 2108 | NGB | Elite line |
| 4 | CP 6 | NGB | Elite line | 49 | ZM 2938 | NGB | Elite line |
| 5 | CP 11 | NGB | Elite line | 50 | ZM 2943 | NGB | Elite line |
| 6 | CP 12 | NGB | Elite line | 51 | ZM 2946 | NGB | Elite line |
| 7 | CP 102 | NGB | Elite line | 52 | ZM 2954 | NGB | Elite line |
| 8 | CP 305 | Malawi | Elite line | 53 | ZM 2966 | NGB | Elite line |
| 9 | CP 399 | Malawi | Elite line | 54 | ZM 2969 | NGB | Elite line |
| 10 | CP 411 | Malawi | Elite line | 55 | ZM 2999 | NGB | Elite line |
| 11 | CP 414 | Malawi | Elite line | 56 | ZM 3003 | NGB | Elite line |
| 12 | CP 418 | Malawi | Elite line | 57 | ZM 3064 | NGB | Elite line |
| 13 | CP 421 | Malawi | Elite line | 58 | ZM 3080 | NGB | Elite line |
| 14 | CP 426 | Malawi | Elite line | 59 | ZM 4710 | NGB | Elite line |
| 15 | CP 436 | Malawi | Elite line | 60 | ZM 5419 | NGB | Elite line |
| 16 | CP 479 | Malawi | Elite line | 61 | Mtilizi | UNZA | Elite line |
| 17 | CP 570 | Malawi | Elite line | 62 | Bgene | Farmers | Landrace |
| 18 | CP 601 | Malawi | Elite line | 63 | Chiko | Farmers | Landrace |
| 19 | CP 633 | Malawi | Elite line | 64 | Chimponongo | Farmers | Landrace |
| 20 | CP 645 | Malawi | Elite line | 65 | Geneb | Farmers | Landrace |
| 21 | CP 698 | Malawi | Elite line | 66 | Kapita | Farmers | Landrace |
| 22 | CP 732 | Malawi | Elite line | 67 | Kapita black | Farmers | Landrace |
| 23 | CP 753 | Malawi | Elite line | 68 | Kapita North | Farmers | Landrace |
| 24 | CP 1769 | Malawi | Elite line | 69 | Chiparamba | Farmers | Landrace |
| 25 | CP 2223 | Malawi | Elite line | 70 | Kapita local | Farmers | Landrace |
| 26 | CP 2231 | Malawi | Elite line | 71 | Lute | Farmers | Landrace |
| 27 | CP 2232 | Malawi | Elite line | 72 | Lutechipata | Farmers | Landrace |
| 28 | CP 2863 | Malawi | Elite line | 73 | Lutembwe chipata | Farmers | Landrace |
| 29 | CP 2980 | Malawi | Elite line | 74 | Makulu | Farmers | Landrace |
| 30 | CP 3067 | Malawi | Elite line | 75 | Mount | Farmers | Landrace |
| 31 | CP 3413 | Malawi | Elite line | 76 | Muz | Farmers | Landrace |
| 32 | CP 3420 | Malawi | Elite line | 77 | BB X SC13(1) | UNZA | Mutant line |
| 33 | CP 3422 | Malawi | Elite line | 78 | BB10-4-2-5 | UNZA | Mutant line |
| 34 | CP 3423 | Malawi | Elite line | 79 | BB14-16-2-2 | UNZA | Mutant line |
| 35 | CP 3425 | Malawi | Elite line | 80 | BB3-9-7-5 | UNZA | Mutant line |
| 36 | 82E -16 | IITA | Elite line | 81 | BB8-1-5-2 | UNZA | Mutant line |
| 37 | Bubebe | UNZA | Elite line | 82 | CP2 X SC 103(1) | UNZA | Mutant line |
| 38 | IT82E-16 | IITA | Elite line | 83 | LT11-3-3-12 | UNZA | Mutant line |
| 39 | L4 X L3(1) | NGB | Elite line | 84 | LT16-7-2-5 | UNZA | Mutant line |
| 40 | L8 X L9 | NGB | Elite line | 85 | LT4-2-4-1 | UNZA | Mutant line |
| 41 | LIO X L7(1) | NGB | Elite line | 86 | LT3-8-4-1 | UNZA | Mutant line |
| 42 | Msandile | UNZA | Elite line | 87 | LT4-2-4-14 | UNZA | Mutant line |
| 43 | Namuseba | UNZA | Elite line | 88 | LTII-3-3-13 | UNZA | Mutant line |
| 44 | Sudan 1 | Malawi | Elite line | 89 | LTII-5-2-2 | UNZA | Mutant line |
| 45 | ZM 1790 | NGB | Elite line | 90 | MSI-8-1-4 | UNZA | Mutant line |

NGB=The National Gene Bank, UNZA=The University of Zambia, all farmers were from Zambia

Note: All accessions from smallholder farmers were from Zambia.
