# Supplementary figures and images for: Assessing the genetic diversity of cowpea [Vigna unguiculata (L.) Walp.] germplasm collections using phenotypic traits and SNP markers
Source: BMC Genet. 2020 Sep 18;21:110. doi: 10.1186/s12863-020-00914-7 (PMC7501654; doi:10.1186/s12863-020-00914-7)

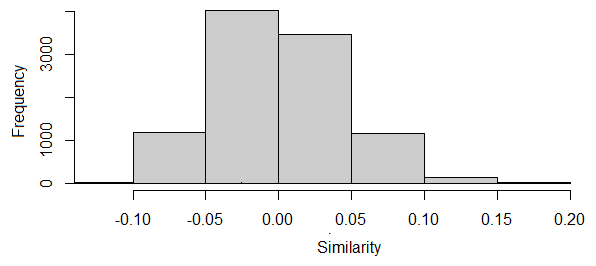


Additional file 4. The Mantel test between phenotypic and genotypic matrices

Supplement: Supplementary file 4 — Additional file 4. The Mantel test between phenotypic and genotypic matrices. [file 12863_2020_914_MOESM4_ESM.docx]

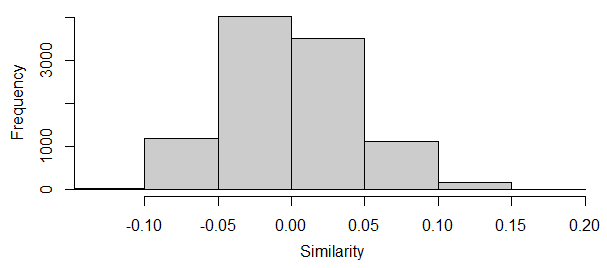


Additional file 5. The Mantel test between phenotypic and joint matrices

Supplement: Supplementary file 5 — Additional file 5. The Mantel test between phenotypic and joint matrices. [file 12863_2020_914_MOESM5_ESM.docx]

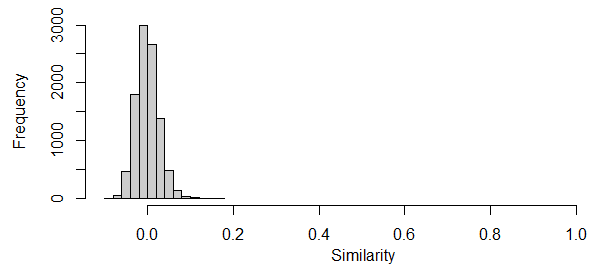


Additional file 6. The Mantel test between genotypic and joint matrices

Supplement: Supplementary file 6 — Additional file 6. The Mantel test between genotypic and joint matrices. [file 12863_2020_914_MOESM6_ESM.docx]

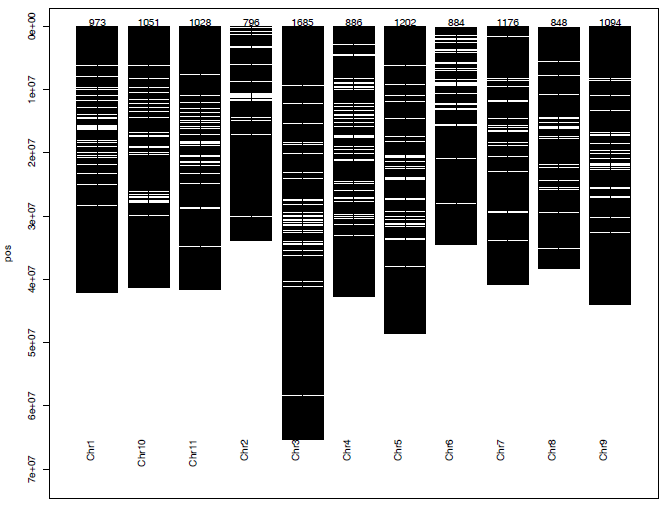


Additional file 7. Chromosomal distribution of the 14,166 SNPs used to evaluate 90 cowpea genotypes

Supplement: Supplementary file 7 — Additional file 7. Chromosomal distribution of the 14,166 SNPs used to evaluate 90 cowpea genotypes. [file 12863_2020_914_MOESM7_ESM.docx]
